# Primary Pulmonary Amebiasis Complicated with Multicystic Empyema

**DOI:** 10.1155/2016/8709347

**Published:** 2016-07-10

**Authors:** Ali Zakaria, Bayan Al-Share, Khaled Al Asad

**Affiliations:** Istishari Hospital, Department of Internal Medicine, Division of Pulmonology, Amman 11183, Jordan

## Abstract

Amebiasis is a parasitic infection caused by the protozoan* Entamoeba histolytica*. While most infections are asymptomatic, the disease could manifest clinically as amebic dysentery and/or extraintestinal invasion in the form of amebic liver abscess or other more rare manifestations such as pulmonary, cardiac, or brain involvement. Herein we are reporting a case of a 24-year-old male with history of Down syndrome who presented with severe right side pneumonia complicated with multicystic empyema resistant to regular medical therapy. Further investigation revealed a positive pleural fluid for* E. histolytica* cysts and trophozoites. The patient was diagnosed with primary pleuropulmonary amebiasis and he responded promptly to surgical drainage and metronidazole therapy. In patients from endemic areas all physicians should keep a high index of suspicion of amebiasis as a cause of pulmonary disease.

## 1. Introduction

Amebiasis occurs worldwide; it is estimated that approximately 40 to 50 million people develop colitis or extraintestinal disease annually with 40,000 deaths [1]. The prevalence is disproportionately increased in developing countries because of poor socioeconomic conditions and sanitation levels. In most cases the infections are asymptomatic yet; it can manifest clinically as intestinal disease or rarely as extraintestinal invasion in the form of liver abscess, pleuropulmonary, cardiac, or brain disease. The lungs are the second most common site of extraintestinal infection, with pleural invasion accounting for 2-3% of patients with invasive disease.

## 2. Case Presentation

The patient is a 24-year-old male with a history of Down syndrome who was brought to our hospital from Yemen by his brother after 10 days of fever (39.5°C), productive cough, shortness of breath, and right side chest pain. He developed the symptoms after few days of being found eating a sandwich that he hid in an organic fertilized soil of his house backyard. He was brought to our hospital in Jordan after he failed a course of moxifloxacin for assumed diagnosis of community acquired pneumonia.

On presentation he was still complaining of same symptoms, and he denied any jaundice, abdominal pain, nausea, vomiting, or diarrhea. On physical examination he was alert, oriented, sweaty, and in mild respiratory distress. His vital signs were as follows: temperature 38.3°C; pulse 110 bpm; respiratory rate 26 bpm; blood pressure 90/45 mmHg; and O_2_% of 82% on room air. He had cracked lips, protruded tongue, erythematous, and dry mucous membranes of the oropharynx. Chest exam revealed significant decreased air entry and tactile vocal fremitus with crackles and minimal wheezes on the right side and normal heart sounds with no murmurs or rubs or gallops. His abdomen was soft and nontender with no evidence of organomegaly.

**Figure 1:**
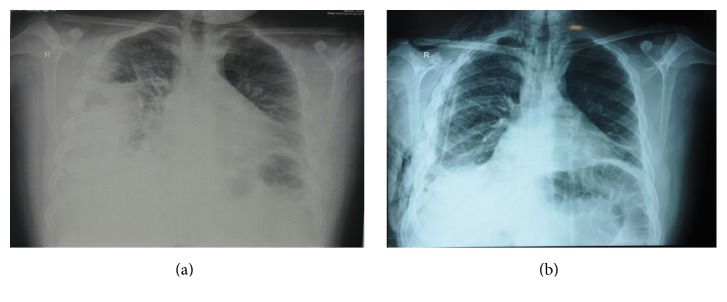
(a) Preoperative chest X-ray shows right sided obliteration of costophrenic angle and displaced right lung. (b) Postoperative resolution of the empyema (note chest tube).

**Figure 2:**
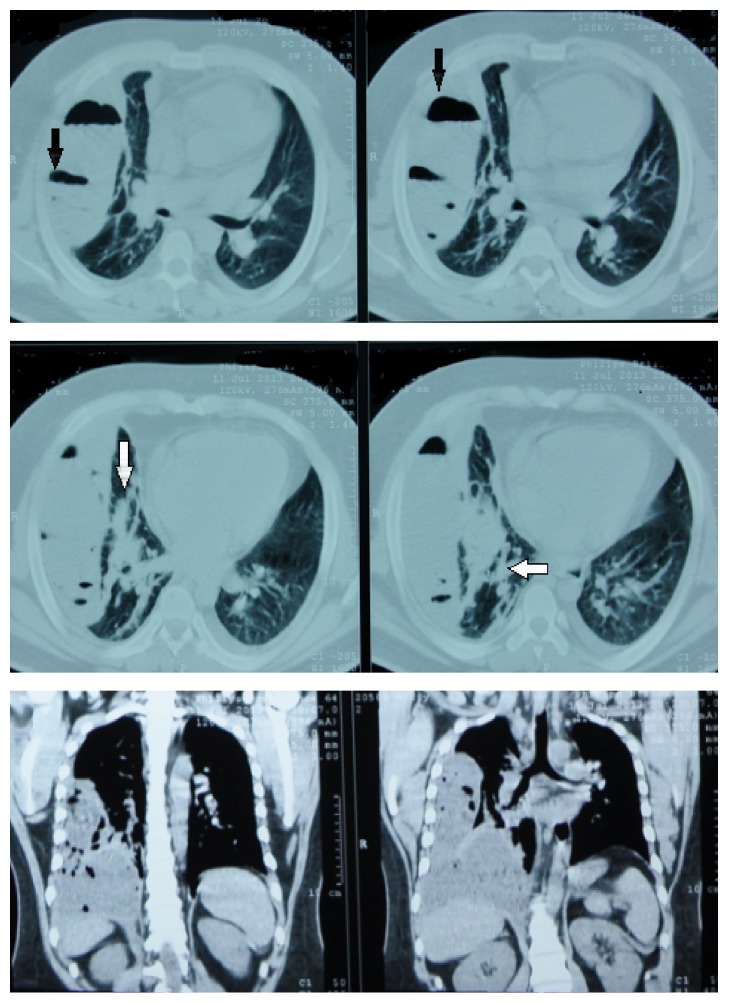
Chest CT scan right sided pleural effusion with pocket mainly in lateral aspect and in the oblique fissure, multiple gas bubbles with air fluid levels (black arrow), and partial atelectasis of right middle and lower lobes that are medially displaced (white arrow).

He was immediately resuscitated with IVF bolus and oxygen supplementation; his chest X-ray revealed right sided obliteration of costophrenic angle and displaced right lung (Figure 1). CT scan showed right sided pleural effusion with pocket mainly in lateral aspect and in the oblique fissure, multiple gas bubbles with air fluid levels, and partial atelectasis of right middle and lower lobes that are medially displaced (Figure 2). Abdomen ultrasound and CT scan showed no evidence of obvious gross liver pathology.

He was diagnosed with right sided pneumonia complicated with multicystic empyema, and he underwent thoracotomy with drainage, decortication, and chest tube placement. Light microscopic examination of both pleural fluid and bronchoalveolar lavage sample revealed* Entamoeba* cysts and trophozoites. Microbiology revealed negative acid-fast stain or fungal infection. Aerobic and anaerobic blood culture showed no growth.

Colonoscopy was done and biopsy revealed mixed inflammatory infiltrate suggestive of infection with no demonstrable amoeba. And stool analysis ×3 was also negative for* Entamoeba* cyst or trophozoites. Serological test was not done due to unavailability.

The patient was treated with metronidazole 750 mg three times daily; luminal agent was not given due to unavailability in Jordan. His clinical condition significantly improved after 48 hours of antibiotic treatment. On one-month follow-up visit he was free of symptoms, and he returned back to Yemen.

## 3. Discussion

Amebiasis is defined by the World Health Organization (WHO) and Pan American Health Organization (PAHO) as a parasitic infection with the protozoan* Entamoeba histolytica* regardless of symptomatology. It is considered the third most common parasitic infection worldwide with around 500 million infections per year and a leading cause of parasite-related mortality with over 100,000 deaths annually. This protozoal infection has an especially high prevalence in subtropical and tropical countries where poor socioeconomic and sanitary conditions predominate, while in resource-rich nations infections may be seen in travelers to and emigrants from endemic areas [2–5].

The majority of* Entamoeba* infections are asymptomatic. Factors that influence whether infection leads to asymptomatic or invasive disease include the* E. histolytica* strain and host factors such as genetic susceptibility, age, and immune status, where young age, pregnancy, corticosteroid treatment, malignancy, malnutrition, and alcoholism are considered risk factors for severe disease [4].

Clinical manifestation of amebiasis generally occurs in the form of intestinal involvement as acute or subacute colitis, with symptoms range from mild diarrhea to severe dysentery producing abdominal pain, diarrhea, and bloody stools, to fulminant amebic colitis. It can also present as extraintestinal disease in the form of amebic liver abscess and even more rare as pulmonary, cardiac, and brain involvement.

Pleuropulmonary complications (i.e., pleural effusion, lung abscess, and, rarely, pleural empyema) are the second most frequent extraintestinal complication; they occur in 7–20% of patients with amebic liver abscesses and in 2-3% of those with invasive disease [6]. The presentation of pleuropulmonary amebiasis is variable and depends on the type of pulmonary involvement whether it is primary simulating bronchopneumonia or tuberculosis or secondary to rupture giving the characteristic suppurative syndrome. The most common symptoms include pain, cough, hemoptysis, and dyspnea. The pain may be pleuritic or localized to the right upper quadrant. Cough can be nonproductive but more often is associated with expectoration of material ranging from small amounts of sputum to large amounts of amebic pus. If a hepatobronchial fistula develops, the patient may expectorate necrotic material that can include liver abscess contents; such material may have a reddish brown or “anchovy sauce” appearance [1].

The theoretical mechanisms of thoracic amebiasis are as follows. First, the infection usually spreads to the lung by direct rupture of an amoebic liver abscess through the diaphragm. Second, the infection may disseminate to the thorax directly from the primary intestinal lesion through hematogenous or lymphatic spread. And finally, inhalation of dust containing cysts of* E. histolytica* is also a hypothetical route (which is the most probable route in our case) [6, 7].

Pleuropulmonary amebiasis is easily confused with other illnesses which makes the differential diagnosis rather a complex one, involving and not limited to (1) pulmonary TB, (2) bacterial lung abscess, (3) carcinoma of the lung, and (4) in endemic areas malaria and schistosomiasis considered common causes of parasitic deaths that can present with unremitting fevers and hepatic or lung disease [8, 9].

The diagnosis of pleuropulmonary amebiasis may be supported by the clinical manifestation and radiographic imaging such as homogenous opacity or cavitating lesion most commonly involving the right lower and middle lobes, elevated right hemidiaphragm, basilar pulmonary infiltrates with areas of focal atelectasis, and pleural effusions. In the setting of suggestive findings on imaging studies, confirmatory serologic or antigenic testing should be pursued and perhaps supplemented with stool microscopy or antigenic testing of stool [10, 11].

Light microscopic examination can often identify characteristic trophozoites and cysts through direct, concentrated, and/or permanently stained smears. Keeping in mind that the organisms may appear intermittently, specimens from patients with disseminated disease may not contain cysts and trophozoites despite repeated examinations [5]. Immunological tests such as indirect hemagglutination assay (IHA) and enzyme-linked immunosorbent assay (ELISA) for* E. histolytica* antibodies are characterized by high sensitivity. The primary disadvantage of serologic tests is that they cannot distinguish between past and current infection unless IgM is detected; IgM antibodies to* E. histolytica* are short-lived and rarely detected. In contrast, IgG antibodies are long-lived but highly prevalent in endemic settings. New serologic tests based on recombinant* E. histolytica* antigens have been developed; such assays may be especially useful in endemic areas [5, 12].

In general, amebic pleural effusions should be aspirated. Drained pleural effusions resolve rapidly with drainage and antimicrobial therapy, which consists of metronidazole (750 mg orally three times daily for 7 to 10 days) or alternatively tinidazole (2 g once daily for five days) [13]. Most patients respond to a single course of treatment with resolution of symptoms before the end of therapy. In rare cases, a second course is needed because of failure to achieve complete resolution after the initial regimen. Treatment with a luminal agent such as paromomycin (25–30 mg/kg/day orally in three divided doses for seven days), diiodohydroxyquin (650 mg orally three times daily for 20 days), or diloxanide furoate (500 mg orally three times daily for 10 days) to eliminate intraluminal cysts is also warranted.

The mortality rate of amebic pleural empyema is as high as 16%, which can increase to 42% due to the rupture of a hepatic abscess into the pleural space. Empyema requires chest tube thoracostomy and decortication to prevent recurrence and chronic infection [8, 10, 14].

In conclusion, pleuropulmonary amebiasis is the second most frequent extraintestinal complication that can be easily treated with drainage and antimicrobial therapy. Inhalation of dust containing cysts of* E. histolytica* is a possible route of primary infection. In patients from endemic areas all physicians should keep a high index of suspicion of amebiasis as a cause of pulmonary disease.
